# Efficacy and safety of inhaled ambroxol solution in improving sputum of lower respiratory tract infection in children: a multicenter, randomized, double-blind, placebo-controlled trial

**DOI:** 10.1186/s12890-025-03845-0

**Published:** 2025-08-08

**Authors:** Baoping Xu, Peng Han, Yunxiao Shang, Huanji Cheng, Zhiying Han, Lin Zhao, Shaoru He, Min Lu, Enmei Liu, Kunling Shen

**Affiliations:** 1 Respiratory Department, Beijing Children’s Hospital, Capital Medical University, China National Clinical Research Center of Respiratory Diseases, National Center for Children’s Health, Beijing, China; 2 Department of Respiratory, Shenzhen Children’s Hospital, Shenzhen, China; 3 Department of Pediatrics, Shengjing Hospital of China Medical University, Shenyang, China; 4 Department of Pediatric Pulmonology, The First Hospital of Jilin University, Changchun, China; 5 Department of Respiratory Medicine, Shanxi Children’s Hospital, Taiyuan, China; 6 Department of Pediatrics, The Second Affiliated Hospital of Kunming Medical University, Kunming, China; 7 Department of Pediatrics, Guangdong Provincial People’s Hospital, Guangdong Academy of Medical Sciences, Guangzhou, China; 8 Department of Respiratory Medicine, Shanghai Children’s Hospital, Shanghai Jiao Tong University, Shanghai, China; 9 Ministry of Education Key Laboratory of Child Development and Disorders, Chongqing Key Laboratory of Pediatrics, Department of Respiratory Medicine, National Clinical Research Center for Child Health and Disorders, Children’s Hospital of Chongqing Medical University, Chongqing, China; 10 Beijing Children’s Hospital, Capital Medical University, No.56 Nanlishi Road, Xicheng District, Beijing, 100045 China

**Keywords:** Ambroxol, Sputum, Children, Lower respiratory tract infections

## Abstract

**Objective:**

To evaluate the efficacy and safety of inhaled ambroxol solution in improving sputum of lower respiratory tract infections (LRTIs) in children.

**Study Design:**

This study was a randomized, double-blind, parallel-group, placebo-controlled, multicenter trial. The patients were administered inhaled ambroxol or a placebo twice a day for 7 days. And researchers collected efficacy and (or) safety indicators every day during the course.

**Results:**

A total of 236 children were randomly assigned to receive ambroxol or placebo (1:1). At all visit points after the medication, the mean difference of cough score with the baseline between the two groups was statistically significant (P < 0.05). Compared with the baseline, the phlegm-sound scores in the throat of the experimental group decreased more on the 1st, 2nd, and 3rd days after administration (P < 0.05). But there was no difference in pulmonary rale scores. The occurrence of adverse events in the experimental group was lower (21.37% vs. 35.59%, P = 0.021), and the incidence of adverse reactions was similar between the two groups (2.56% vs. 5.08%, P = 0.499).

**Conclusion:**

Inhaled ambroxol solution could improve the sticky sputum symptoms in children with LRTIs and is safe in clinical application. Further research is needed to confirm these findings.

**Trial registration:**

The study was retrospectively registered on June 14, 2023, at https://www.chictr.org.cn/ under the number ChiCTR2300072466.

## Introduction

Lower respiratory tract infections (LRTIs) are a substantial public health problem and the leading cause of morbidity and mortality in people of all ages. Cough and expectoration are the most common symptoms of LRTI. The perceived importance of mucus in the pathophysiology of LRTIs have led to the development of drugs intended to treat airway mucus hypersecretion [1]. Essentially, the therapy is mainly aimed at the pathophysiology of mucus hypersecretion, from inhibiting the secretion of specific endogenous inflammatory factors to promoting mucus dissolution to assist expectoration. Airway mucus is an important part of the respiratory defense barrier, and the important component is mucin. The expression of mucin-5AC(MUC5AC) is remarkably increased by the action of inflammatory mediators and cytokines, which leads to the retention of mucus or the formation of a mucus plug and makes the condition worse.

Ambroxol, the eighth active metabolite of bromhexine, is a pectolytic and muco-active over-the-counter agent primarily used to treat respiratory diseases associated with sticky sputum. Ambroxol can facilitate airway mucosa cilia clearance by reducing the glycosaminoglycans level in bronchoalveolar lavage fluid and MUC5AC mRNA level in lung tissues [2]. It can also increase ciliary beat frequency to optimize mucociliary clearance [3]. And in-vitro it selectively inhibits Na^+^ absorption by airway epithelium, thereby increasing water in airway surface fluid and reducing mucus viscosity [4]. In addition to mucolytic action, Ambroxol has antioxidant and anti-inflammatory properties in vitro [5] and in vivo [6]. Because of the effects of anti-inflammatory and pectolytic activity, ambroxol is widely used in clinic.

It was already 20 years of ambroxol clinical application in China, but it was mainly in oral liquid, sustained-release tablets, and injections. Somewhere, ambroxol hydrochloride injection was also administered in the form of aerosol inhalation sometimes, especially for children, which was off-label medication. However, aerosol inhalation therapy is one of the most important treatments for respiratory diseases especially for children. Compared with oral, intramuscular injection, and intravenous administration, aerosol inhalation therapy, making drugs directly acting on the target organ, has a rapid onset, better effect, fewer systemic adverse reactions, and no need for deliberate cooperation by patients which is more considerable for younger children [7–9]. Until now, little was known about the potential of inhaled ambroxol for children. Thus, our aim was to evaluate the efficacy and safety of ambroxol hydrochloride solution in the treatment of children with LRTIs.

## Methods

### Study design and participants

This study was designed as a randomized, double-blind, parallel-group, placebo-controlled, multicenter trial. This trial had nine institution hospitals located in different areas of China. Patients were prospectively enrolled and followed up from September 2013 through February 2014. To ensure the quality of study, all trial centers were managed by a major investigator. All procedures performed in studies involving human participants were in accordance with the ethical standards as laid down in the 1964 Declaration of Helsinki and its later amendments. This trial was approved by the Ethics Committee of Beijing Children’s Hospital affiliated with Capital Medical University (the ethical approval number: [2013]−8-1). The study was retrospectively registered on June 14, 2023, at https://www.chictr.org.cn/ under the number ChiCTR2300072466. All participants or authorized surrogates would be given a detailed explanation of this trial. The guardian of these children voluntarily signed a written informed consent form, meanwhile, the children whose age more than 10 years old also required to sign by themself.

Inpatients aged of 6 months to 12 years with a diagnosis of LRTIs (included acute bronchitis and pneumonia) were eligible for this study. The diagnostic criteria of acute bronchitis included: (1) dry cough or cough with sputum, fever, and symptoms like loss of appetite, vomiting, or diarrhea; (2) pharyngeal congestion, coarse breath sounds with non-fixed, scattered dry and moist rales; (3) chest X-ray may show normal findings or increased lung markings and deepened hilar shadows [10, 11]. The diagnostic criteria of pneumonia included: (1) onset may be acute or gradual, with symptoms such as fever, cough, and sputum production, (2) early pulmonary signs may be subtle, with coarse breath sounds or slightly reduced breath sounds. Later, medium and coarse moist rales and dullness on percussion may appear, and fine moist rales or crackles may develop after a few days, (3) in bacterial infections, white blood cell count and neutrophils are elevated, while in viral pneumonia, white blood cell count is normal or decreased and chest X-ray typically shows increased lung markings and may reveal patchy shadows [10, 11]. Additionally, children were required to have sticky phlegm and expectorating sputum difficulty that cough score were more than 2 points.

Patients were excluded if they met one of the following criteria: (1) Patients were allergic to more than one kind of substance contained in the drug; (2) Patients with upper respiratory tract infections; (3) Patients had wheezing symptoms (requiring bronchodilator treatment); (4) Patients with severe pneumonia, bronchiolitis, asthma, bronchiectasis, and pulmonary fibrosis; (5) Patients had symptoms of respiratory depression or tissue hypoxia; (6) Patients with severe primary diseases of cardiovascular, cerebrovascular, liver, kidney and hematopoietic system; (7) Patients with drug dependent or mental disorders; (8) Patients had participated in or were participating in other clinical trials within 3 months.

Withdrawal criteria:1) Patients not suitable to continue the research because of the severity of their disease; 2) Patients withdraw from the trial by themselves.

### Randomization and masking

The dynamic randomization method was used to randomize subjects into groups through the central randomization system. The stratification factor was age (6 months to 2 years old; 2 to 12 years old). The random number table was provided by statistical professionals in the Department of Health Statistics, Naval Medical University. The preparation of drug blinding and emergency letters was completed by personnel unrelated to the clinical trial. The central randomization system administrator sent an invitation code to the researchers in each center, and the researcher’s evaluation invitation code was registered in the central randomization system.

The specifications of Ambroxol hydrochloride solution for inhalation, which contains ambroxol hydrochloride 15 mg, and placebo both were 2 ml/bottle. The appearance, color, and osmotic pressure were the same and all drugs were produced by Hanmi Pharmaceutical Co., Ltd. and were qualified.

### Procedures

**Fig. 1:**
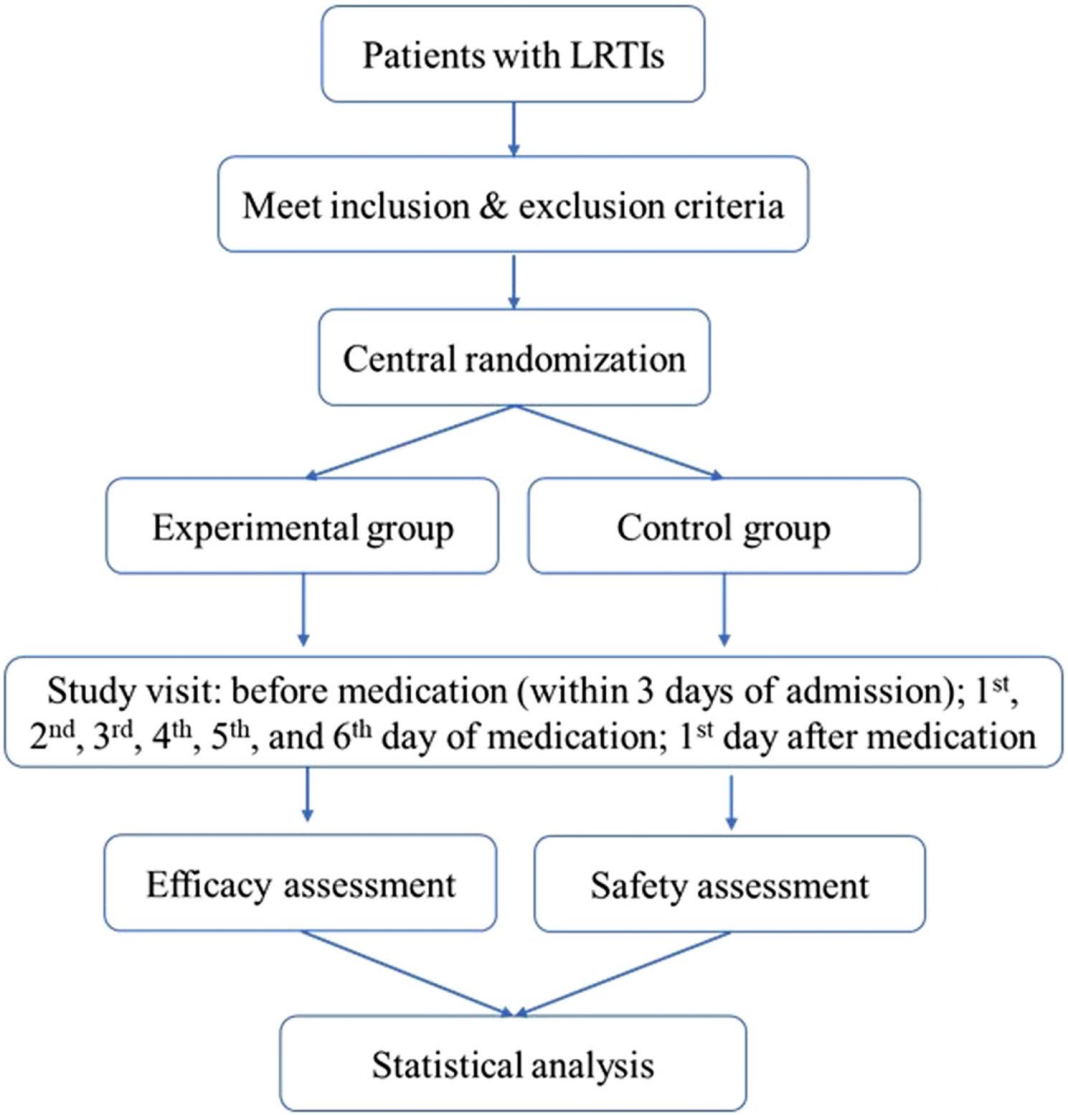
Flowchart of the study

The patients with LRTIs, who fulfill the inclusion and exclusion criteria, were randomized into the experimental or control group in a ratio of 1:1. The placebo and ambroxol solution for the study were treated by aerosol inhalation in a quiet environment through a uniform atomizing drug delivery device, by diluting with 0.9% sodium chloride injection at a ratio of 1:1. The patients were administered 2 times a day with an interval of no less than 6 h during the 7 days course of treatment. All subjects used the doses according to the age (6 months to 2 years old, 1 ml; 2 to 12 years old, 2 ml). The daily nebulized inhalation drugs were uniformly distributed and distributed by authorized nurses to record the use of drugs. The patients were clinically cured and judged by doctors when the symptoms, and signs completely improved. Then stopped the medication and completing the relevant laboratory examinations, the patient was deemed to have completed the trial and was included in the per-protocol set (PPS). The flowchart of the study was shown in Fig. 1.

Drugs were permitted to be used together with ambroxol in our research, including: (1) Antibacterial drugs: If the patients needed to use antibiotics based on their symptoms, signs, and examination results, the investigator can use and adjust medication referring to the “Guiding Principles for the Clinical Application of Antimicrobial Drugs” [12] and guidelines for related diseases [13]. (2) Febrifuge when necessary. (3) The medicine that children must use to treat other diseases, was recorded in detail (times and dosage).

Drugs not permitted, included: (1) Other drugs that can improve the symptom of sputum among patients with LRTIs. (2) β_2_ receptor agonists. (3) Other drugs inhaled by nebulization.

### Definitions

The primary efficacy outcome was the improvement of cough. Secondary efficacy outcomes included the improvement of phlegm sound in the throat and the improvement of rales in the lung. The severity of symptoms/signs was assessed on a scale of 0 to 3 and recorded at each visit point. The score evaluation criteria were shown in Table 1 [14–16]. The baseline score of cough and phlegm sound in the throat were evaluated within 24 h before enrollment. The baseline score of rales in the lung was based on the auscultation when the children were enrolled.

**Table 1:** The criteria for evaluating the severity of symptoms/signs

Symptoms/signs	0	1	2	3
	Normal	Mild	Moderate	Severe
Cough	Without	Intermittent, not affect daily life	Between mild and severe	Frequent, coughing day and night, affecting sleep
Phlegm sound in the throat	Without	Occasional	Common	Frequent
Rales in the lung	Without	Little rales	Medium rales	Lots of rales

Safety observation outcomes: (1) adverse events; (2) vital signs (temperature, breath rate, heart rate, blood pressure); (3) red blood cell, white blood cell, hemoglobin, platelet, urine leukocyte, urine erythrocyte, urine protein, glucose, alanine aminotransferase, aspartate aminotransferase, total bilirubin, alkaline phosphatase, γ-glutamyl transpeptidase, blood urea nitrogen and creatinine; (4) 12-lead electrocardiogram (for the patients over 3 years old).

### Statistical analyses

To calculate the sample size in this study, we conducted an adult preliminary experiment to compare the improvement of cough scores on the third day after medication. In the preliminary experiment, the difference from the baseline in the experimental group was 0.83 with a standard deviation (SD) of 0.65. In the control group, the difference was 0.52 (SD = 0.59). According to a one-sided type I error of 0.025 and 80% power to detect the improvement of symptoms/signs in treatment by ambroxol compared with placebo, the sample size was at least 64 patients in each group. Furthermore, to meet the requirements of the drug registration management measures in China and consideration of the 20% dropout rate, we finally decided that the sample size of each group was 120 patients, and the total sample size was 240 patients.

The baseline analysis used the data of the intention-to-treat set (ITS). Efficacy data were analyzed using both the ITS and PPS. The intention-to-treat population included all randomized patients who received at least 1 dose of the study drug. The per-protocol population included all randomized patients who met all inclusion criteria, received all doses of the study drug, and did not have serious deviations from the protocol. The data of adverse events and adverse reactions were analyzed through a safety set (SS) in which patients had taken the drug and had at least one safety evaluation record.

We report the number and percentage of patients for categorical variables, the median and interquartile range for continuous variables with nonnormal distribution, and the mean and SD for those with normal distribution. Categorical variables were compared using the chi-square test or the Fisher exact test. Continuous variables were compared using the t-test or the non-parametric Mann-Whitney test. We calculated 95% confidence intervals (95% CI) for differences in outcome rates and medians. The comparison of the two groups before and after treatment was performed using the rank sum test, and the comparison within the group before and after treatment was performed using the signed-rank test. All tests were 2-tailed and the significance was set at 0.05. All analyses were performed with SAS 9.3.

## Results

### Baseline characters of participants

**Fig. 2:**
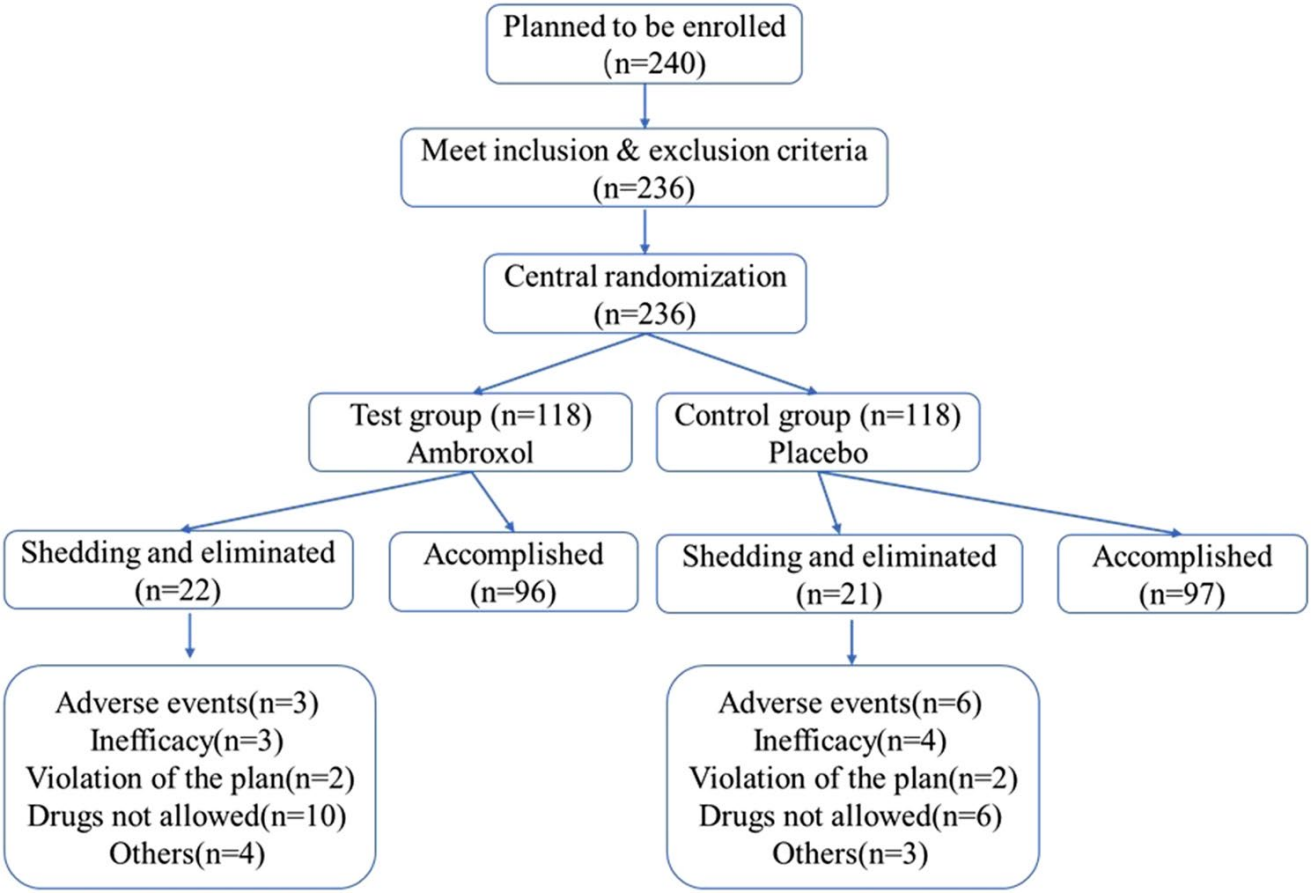
The flowchart of the subjects enrolled, eliminated and shedding

A total of 240 cases were planned to be enrolled, and 236 children were randomly assigned to receive ambroxol (118) or placebo (118). There were 23 cases of shedding, including 10 cases in the experimental group (shedding rate 8.47%); 13 cases in the control group (shedding rate 11.02%). 20 cases were eliminated, of which 12 were eliminated in the experimental group (elimination rate 10.17%); 8 cases were eliminated in the control group (elimination rate 6.78%). Figure 2 showed the flowchart of the subjects enrolled, eliminated, and shedding in the trial. 235 cases were entered into ITS analysis (117 cases in the experimental group, 118 cases in the control group). 202 cases entered PPS analysis (100 cases in the experimental group and 102 cases in the control group). 235 cases were entered in SS analysis (117 cases in the test group and 118 cases in the control group).

There was no statistically significant difference between the two groups in demographic characteristics, disease distribution, past medical history, treatment history, and combined medication history (all *P* values > 0.05, shown in Table 2).

**Table 2:** Characteristics of subjects in the study

	Experimental group (*n* = 117)		Control group (*n* = 118)		*P* value
Demographic					
Male, n(%)	71	(60.68)	63	(53.39)	0.293
Median age, years (IQR)	4	(3–6)	4	(2–7)	0.711
Han nationality, n (%)	112	(95.73)	113	(95.76)	1.000
Mean height, cm (SD)	108.14	(22.62)	106.31	(21.28)	0.525
Mean weight, Kg (SD)	19.68	(9.76)	20.43	(11.11)	0.585
Disease distribution					
Acute bronchitis, n(%)	16	(13.68)	18	(15.25)	0.853
Pneumonia, n (%)	101	(86.36)	100	(84.75)	0.853
Another disease, n(%)	1	(0.85)	0	(0.00)	0.498
Allergy history, n (%)	17	(14.53)	20	(16.95)	0.721
Past medical history, n (%)	36	(30.77)	27	(22.88)	0.187
Treatment history, n(%)	83	(70.94)	90	(76.92)	0.372
Combined medication history, n(%)	94	(80.34)	95	(80.51)	1.000

### Efficacy analysis

#### Primary efficacy outcome

**Fig. 3:**
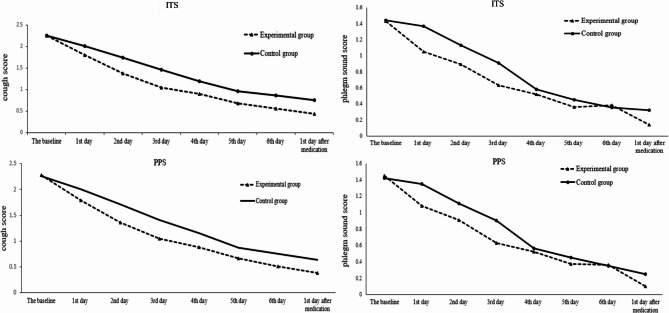
Changes of cough score (mean) and phlegm sound score in the throat (mean) in the two groups before and after treatment. Legend for Figure 3: ITS: intention-to-treat set. PPS: per-protocol set. The P value of the mean difference with the baseline between the two groups at all visit points was less than 0.05

The mean difference was compared between the values of cough score at each visit point and the baseline of the two groups, using ITS and PPS analysis. It was observed that cough scores were statistically significantly lower in the experimental group. At all visit points after the medication, the mean difference with the baseline between the two groups was statistically significant (*P* < 0.05, shown the detail in Table 3). And this difference can be detected on the first day after medication (shown in Fig. 3). The result of ITS analysis was consistent with the PPS.

**Table 3:** The comparison of cough scores between two groups in ITS and PPS

	ITS					PPS				
	Experimental group (*n* = 117)		Control group (*n* = 118)		*P* value	Experimental group (*n* = 100)		Control group (*n* = 102)		*P* value
	Average cough score	Difference between baseline	Average cough score	Difference between baseline		Average cough score	Difference between baseline	Average cough score	Difference between baseline	
The baseline (SD)	2.26 (0.44)	0.00	2.25 (0.44)	0.00	0.8526	2.29 (0.46)	0.00	2.26 (0.44)	0.00	0.6899
1 st day of medication (SD)	1.80 (0.48)	0.46	2.01 (0.58)	0.25	0.0009	1.79 (0.48)	0.50	2.00 (0.56)	0.26	0.0010
2nd day of medication (SD)	1.38 (0.54)	0.89	1.74 (0.63)	0.52	< 0.0001	1.37 (0.54)	0.92	1.71 (0.62)	0.56	< 0.0001
3rd day of medication (SD)	1.05 (0.51)	1.21	1.46 (0.66)	0.80	< 0.0001	1.05 (0.52)	1.24	1.41 (0.63)	0.85	< 0.0001
4th day of medication (SD)	0.90 (0.50)	1.37	1.19 (0.71)	1.09	0.0023	0.88 (0.52)	1.41	1.15 (0.67)	1.12	0.0057
5th day of medication (SD)	0.68 (0.57)	1.58	0.96 (0.72)	1.30	0.0069	0.66 (0.57)	1.63	0.87 (0.66)	1.39	0.0209
6th day of medication (SD)	0.56 (0.55)	1.71	0.86 (0.75)	1.40	0.0038	0.50 (0.52)	1.79	0.75 (0.68)	1.51	0.0067
1 st day after medication (SD)	0.44 (0.56)	1.82	0.75 (0.71)	1.51	0.0019	0.37 (0.49)	1.92	0.64 (0.59)	1.63	0.0022

*ITS* Intention-to-treat set, *PPS* Per-protocol set

#### Secondary efficacy outcomes

The mean difference was compared between the phlegm sound scores in the throat at each visit point and the baseline of the two groups in ITS and PPS analysis. The experimental group decreased by 0.38, 0.54, and 0.83 on the 1 st day, 2nd day, and 3rd day of medication. Meanwhile, the control group decreased by 0.07, 0.29, and 0.50 respectively. And the difference between the two groups was statistically significant (*P* < 0.05) on the 1 st day, 2nd day, and 3rd day of medication (shown in Fig. 3). The curative effect of the experimental group was better than the control group. This difference was no longer statistically significant from the 4th day of medication. The result of the ITS analysis was consistent with the PPS.

Whether in ITS analysis or PPS analysis, the mean difference in pulmonary rale scores between each visit point and the baseline was not statistically significant between the two groups (*P* > 0.05).

### Safety outcomes

In this study, 117 patients of the experimental group and 118 patients of the control group were entered into SS. The occurrence of adverse events in the control group was significantly higher than that in the experimental group (shown in Table 4). Furthermore, the incidence of adverse reactions was 2.56% in the experimental group and 5.08% in the control group respectively, and the incidence was similar between the two groups (*P* > 0.05).

**Table 4:** The occurrence and comparison of adverse events in the two groups

	The experimental group (*n* = 117)			Control group (*n* = 118)			*P* value
	Patients(*n*)	Periods	Incidence (%)	Patients(*n*)	Periods	Incidence (%)	
Adverse events	25	33	21.37	42	54	35.59	0.021
Mild adverse events*	17	22	14.53	28	35	23.73	0.097
Serious adverse event*	1	1	0.85	0	0	0.00	0.498
Adverse reactions	3	4	2.56	6	6	5.08	0.499

*The criteria for the severity of adverse events: Mild adverse events were usually transient and not affecting normal daily activities. Serious adverse events were affecting normal daily activities

The adverse reactions included wheezing, skin allergy, and abnormal liver function. The patients with adverse reactions to wheezing in the two groups stopped using the drug and then withdrew from the clinical trial. The patients with skin allergy, given the symptomatic treatment, did not suspend the administration and completed the clinical trial. The patients with abnormal liver function stopped taking the medication and did not withdraw from the clinical trial. All of the adverse reactions turned to disappear without sequelae.

Considering that all patients were in the period of illness, the changes in vital signs, laboratory examinations, and other safety indicators might be related to the disease itself. The two groups both had the situations of “normal before treatment, abnormal after treatment” and “abnormal before and after treatment”, most of which had no clinical significance and may not be related to ambroxol. However, several patients who had abnormal laboratory examinations after the treatment had already been reported as adverse events (see Table 4).

## Discussion

It has been already 20 years since the clinical application of ambroxol hydrochloride except for the inhalation form in China [16]. So far, there has been no study about the efficacy and safety of ambroxol hydrochloride solution for inhalation in Chinese children. Our research filled this gap, especially for children with sticky sputum and cough.

Furthermore, our study was the first standardized clinical study of ambroxol hydrochloride solution for inhalation worldwide to enroll children under 6 years old. A total of 9 hospitals participated in the clinical trial lasted for 150 days. The placebo-controlled, double-blind, pharmacokinetic clinical study in 1993 showed no severe adverse events were reported and no drug-induced changes in the clinical laboratory values were observed with 1 g/day of ambroxol [17]. In the process, considering the characteristic of pediatrics, different doses of drugs were given according to the age of the group to reduce the risk of the trial. Moreover, we further reduced the drug risk by strictly controlling the selection criteria in the trial, excluding patients with severe pneumonia, and early withdrawing the study of children with aggravated conditions, certain comorbidities, complications, and special physiological changes.

Ambroxol hydrochloride solution for inhalation is suitable for young children with LRTIs. As for inhalation, ambroxol hydrochloride solution has no smell and does not require deliberate cooperation by patients. The solution for inhalation adopted the blow-fill-seal technology certified by an international authority which is stricter and higher degree of sterility protection [18, 19]. It could reduce the risk of children in the process of inhalation to a certain extent.

The primary action of ambroxol is to improve mucociliary clearance by reducing sputum viscosity, which indirectly alleviates cough by facilitating mucus elimination. The observed reduction in cough scores is likely due to improved mucus clearance rather than a direct antitussive effect. As for the phlegm in the throat, the experimental group improved better than the control group in the early stage of medication. As we all know, there is more sputum in the early stage of LRTIs. Based on the effect of the drug, it is likely to have a more significant therapeutic effect in the early stage of LRTIs. This is consistent with the results of our study.

In addition, there were three aspects for consideration about no difference between the two groups in the comparison of pulmonary rales. Firstly, through the nebulization device, ambroxol hydrochloride solution for inhalation mainly interfered with the sputum in the bronchus and could not directly act on the bronchioles. While the pulmonary rales mainly exist in the bronchioles. Therefore, the effect of reducing the comparison of pulmonary rales was not obvious. Secondly, there wasn’t a detailed description of pulmonary rales in this study. Lastly, the rales required the researchers to auscultate, which was related to the researcher’s subjective judgment and might affect the accuracy of the result. The categorical scoring systems may have limitations in capturing the full spectrum of symptom relief. However, this approach is widely used in clinical practice and has been validated in previous studies for assessing respiratory symptoms in children with LRTIs. Future studies could consider incorporating continuous variables or more sensitive measures to better evaluate symptom improvement.

Recent data analysis has revealed that the mucolytics play an important role in relieving cough symptoms by facilitating the elimination of mucus [20]. And other studies found ambroxol could increase mucociliary transport rates and reduce the elasticity and viscosity of mucus in airways [21, 22]. Moreover, ambroxol can reduce glycosaminoglycans levels in BALF. It also increases surfactant production and alkaline phosphatase secretion and alters ion transport. The research data on inhaled ambroxol are limited in both experimental and clinical settings [2, 23]. The results of the current study may not fully elucidate its complex pharmacological actions. Further research is needed to comprehensively evaluate the mechanisms of action and clinical efficacy of ambroxol.

Ambroxol can increase the concentration of antibiotics in lung tissue, and a significant clinical and radiological improvement was observed in the ambroxol and antibiotics group compared with antibiotics alone [24, 25]. The failure to collect data regarding the use of antimicrobials is a limitation of the study. Drugs such as azithromycin are known to decrease cough and sputum production. Future studies should be focus on this aspect.

The safety of ambroxol has already been well-established based on its use in more than 15,000 patients, with a total patient exposure estimated at 4,789,563 patient years [16]. According to randomized, placebo-controlled trials, ambroxol was well-tolerated during short-term and long-term treatments (up to two years), showing no difference in adverse events compared to the placebo groups [26, 27]. This is consistent with our research results.

Limited by the quantity of sample, not all types of adverse reactions were observed. In our study, the adverse reactions in the experimental group mainly manifested in mild wheezing and mild skin allergies which were consistent with the adverse reactions reported in the market. Now ambroxol hydrochloride solutions for inhalation have already been available. The research is ongoing to collect real-world evidence using validated tools for continuous monitoring of efficacy and safety.

## Conclusion

Inhaled ambroxol solution could improve the sticky sputum symptoms in children with LRTIs and is safe in clinical application. Further research is needed to confirm these findings.

## Data Availability

All data generated or analyzed during this study are included in this published article.
